# Performance comparison of MOF and other sorbent materials in removing key odorants emitted from pigpen slurry

**DOI:** 10.1038/srep31283

**Published:** 2016-08-11

**Authors:** Ezaz Ahmed, Akash Deep, Eilhann E. Kwon, Richard J. C. Brown, Ki-Hyun Kim

**Affiliations:** 1 Department of Civil and Environmental Engineering, Hanyang University, 222 Wangsimni-Ro, Seoul 04763, Korea; 2 Central Scientific Instruments Organisation (CSIR-CSIO), Sector 30 C, Chandigarh, 160030, India; 3 Department of Environment and Energy, Sejong University, Seoul 05006, Republic of Korea; 4 Environment Division, National Physical Laboratory, Teddington, TW11 0LW, UK

## Abstract

A batch-type dynamic headspace (HS) system was used to generate vapor-phase volatile organic compounds (VOCs) from a pigpen slurry sample. Sorptive removal capability of MOF-199 and other sorbents (zeolite (ZL) and activated carbon (AC)) was assessed against a total of 13 slurry-borne odorants ((methyl ethyl ketone (MEK), isobutyl alcohol (i-BuAl), benzene (B), toluene (T), p-xylene (p-X), m-xylene (m-X), o-xylene (o-X), styrene (S), o-cresol (o-C), phenol (PhAl), p-cresol (p-C), indole (ID), and skatole (SK)). Adsorption capacity of MOF-199 and two sorbents, when assessed for the 13 odorants at a 10% breakthrough volume (BTV), was 22.6  ±  42.3, 0.70  ±  1.08, and 11.0  ±  18.3 μg g^−1^, respectively. The adsorption capacity (μg g^−1^) assessed at 10% BTV showed the superiority of MOF-199 towards phenolic and indolic compounds (such as o-C (0.31  ±  0.04), PhAl (61.6  ±  4.98), p-C (140  ±  7.95), ID (27.8  ±  2.23), and SK (63.9  ±  1.55)), demonstrating the feasibility of MOF as sorption media for treating certain nuisance components.

Livestock farming has undergone enormous structural modifications over the past few decades resulting in more animals being raised on large commercial feedlots than ever before^1^. Also, the increased size and yearlong production cycles have led to a growing recognition of the environmental hazards of the industry for both air and water resources^2^. Livestock production is an important source of offensive odors that degrade air quality and has a negative impact on the environment over long distances^3^,^4^,^5^. However, odor nuisance from swine slurry becomes more acute when the slurry is agitated or stirred, i.e., during manure handling^6^,^7^. Therefore, assessment of air quality inside and nearby these operations and the removal or minimization of unpleasant odorants is now a prerequisite to ensure acceptable air quality and proper environmental management^8^.

Increased attention has been paid recently to pollutants emitted from animal feeding operations (AFOs) and wastewater treatment facilities due to their adversary impacts on the environment and on human health^9^,^10^. In a landmark report on air emissions from AFOs, the National Research Council (NRC), USA indicated that emission of odors from AFOs is of major importance at the local level^11^. A number of VOCs including volatile fatty acids (VFAs), phenols, indoles, and skatoles have been detected from various source environments such as wastewater treatment facilities and AFOs^12^,^13^,^14^. A large number of VOCs have been identified in emissions from livestock facilities and in confined spaces, such as swine buildings, including alcohols, aldehydes, amines, esters, ethers, hydrocarbons, ketones, nitrogen-containing compounds, phenols, indoles, other aromatic compounds, terpenes, and sulfur containing compounds^15^,^16^,^17^.

Various sorption methods have long been employed for the removal of VOCs and other pollutants^18^,^19^. The adsorption capacity of sorbent materials mainly depend on pore characteristics such as specific surface area, pore size, and pore size distribution^20^. The pollutants are adsorbed on the surface of a granule, bead, or crystal of adsorbent materials. The adsorbed material is held physically, not particularly strongly, and can be released (desorbed) easily by either heat or vacuum. Thus, it is a challenging issue to develop suitable sorbent materials to efficiently control odorants emitted from various agro-industrial sources at lower cost.

Metal-organic frameworks (MOFs) are a recently identified class of crystalline nanoporous materials built of small metal-containing clusters connected, three-dimensionally, by poly-functional organic ligands^21^,^22^. They exhibit large surface areas and are good candidates for odor removal as they can exhibit wide geometrical and chemical variety and thus may be structurally tuned to trap effectively the molecules requiring removal^23^. Their gas sorption properties have been extensively reported suggesting benefits for many important applications (viz, wide range of gas storage, active filtration, etc.)^24^,^25^. The two most popular MOFs ‘MOF-5 and MOF-177’ cost around $200/kg and $130 K/kg, respectively (http://jclfss.weebly.com/design-and-cost.html). With new developments in the synthesis process and the industrial upscaling of the MOF production, these compounds are expected to be even cheaper^26^. In this work, the adsorption performance of Cu-BTC MOF of various odorants (containing diverse functional groups) was investigated. A batch-type dynamic headspace-based approach was established as a means of generating diverse vapor-phase volatile organic compounds from the liquid slurry sample placed in an impinger. We tested the performance of MOF-199, zeolite (ZL), and activated carbon (AC) to remove volatile odorants emitted from pigpen slurry. The basic characteristics of the adsorbents used in our study are presented in Table 1.

In the quantitative stage of this study, a total of 19 target VOCs (refer to Table 1S in Supplementary Information) was considered throughout the experimental analysis. Note that acronyms are generally used for all target compounds throughout the text. Identical protocols were followed both for sample collection (by a sorbent tube (ST)) and the instrumental detection (by the same GC-mass spectrometry (MS) system equipped with a thermal desorber (TD))^27^. The extracted ion chromatogram (EIC) mode of the MS system was applied to quantify the target VOCs at trace quantities in this study^28^.

## Results and Discussion

### General characteristics of the odorants emitted from slurry

The initial concentration of odorants emitted from the system is governed by air−water partition coefficients (e.g., the ideal Henry’s law behavior)^29^. Subsequently, the emissions reach a steady-state equilibrium stage (non-ideal Henry’s law behavior). At the equilibrium stage emissions occur steadily from the slurry sample^30^.

In our study, quantification of the odorants was performed at the beginning and at the end of each experiment cycle. At first, the concentration levels of odorants partitioned into slurry headspace (HS) were measured by 5 min sampling after an initial purging of 20 min (at a constant flow rate of 100 mL min^−1^). Our experiments of odorant removal were carried out by passing odorants through the sorbent bed for ten consecutive runs to make a total loading volume of of 5 L on the tested sorbent. Immediately after finishing that experiment (or carrying out 10 consecutive runs), another 5 min samples were taken shortly afterwards to check the stability of the odor generation system (slurry) (presented in Table 2). As presented in Table 2A, p-C (589 ± 4.23 ppb) is the most prominent odorant emitted from the slurry, whereas o-C (1.73 ± 0.00 ppb) has the lowest detected concentration. However, the odorants emitted from the HS-sampling in the first experiment cycle (D1) showed the following concentration trend from the highest to the lowest: p-C (589 ± 4.23) > MEK (313 ± 15.0) > PhAl (272 ± 9.30) > SK (188 ± 0.00) > T (104 ± 12.1) > ID (67.7 ± 0.00) > S (53.4 ± 10.8) > B (32.1 ± 7.40) > p-X (29.7 ± 8.81) > i-BuAl (17.2 ± 0.00) > m-X (3.14 ± 0.21) > o-X (1.97 ± 0.52) > o-C (1.73 ± 0.00 ppb). An almost similar concentration pattern (except for B) was observed for two other experiment cycles (D2 and D3).

**Figure 1:**
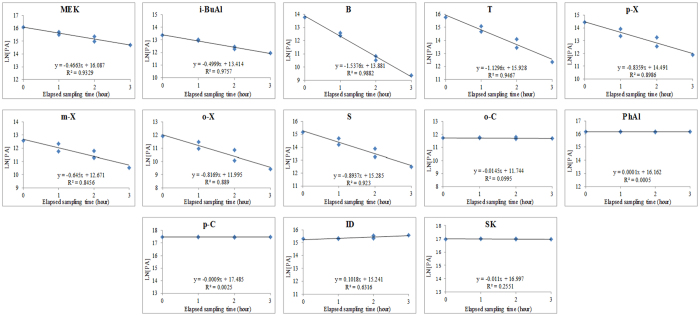
Odorants emitted from the impinger based headspace sampling with respect to elapsed sampling time (hour) over three consecutive days.

In order to learn about the changes in odorant emission rates with respect to storage time, the impinger system loaded with the same slurry sample was analyzed on three consecutive days without disturbing the samples. To assess the temporal trends in odorant emission, we compared the relationship between the natural logarithm function of peak area (LN[PA]) vs. elapsed sampling time (Fig. 1). The loss of highly volatile compounds over time is clearly distinguishable from that of less volatile ones (e.g., o-C, PhAl, p-C, ID, and SK). For instance, the slope values for the concentration change with elapsed sampling time is high and negative for B (−1.5376) compared to almost zero for PhAl (0.0001). This observation may directly reflect the effect of differences in the liquid-gas partitioning behavior of different odorants^30^ and possibly the extent of their hydrophobicity^31^. Due to differences in hydrophobicity, the extraction of volatile compounds can occur more effectively in the first purging cycle, and then the extent of extraction decreases in the subsequent purging cycles. In contrast, semi-volatile compounds (e.g., o-C, PhAl, p-C, ID, and SK) with relatively low hydrophobicity tend to exhibit relatively constant extraction with purging cycles as a large proportion of them remains in the liquid phase and this does not change significantly over time.

Table 2B indicates that the emission rates of most volatile odorants (MEK, i-BuAl, B, T, p-X, m-X, o-X, and S) showed a systematic decrease with time. In contrast, those of phenolic and indolic compounds (o-C, PhAl, p-C, ID, and SK) maintained a consistent emission trend throughout the experiment period. Hobbs *et al*.^32^ also observed decreased emission rates for the lighter volatile odorants emitted from swine slurry with a function of storage period. The changes in odorants emission trends, as observed in our study, can thus be attributed to the differences in the physicochemical properties of odorants released from the slurry samples^33^,^34^.

### Breakthrough volume of all target odorants against MOF-199

As aforementioned, sorbents were tested by loading 0.5 L of dynamic headspace samples for 10 consecutive runs (a total loading volume of 5 L). The unadsorbed portion of VOCs eluting from the adsorbent materials was then directly collected on a 3-bed sampling ST to assess the sorptive losses of VOCs. It was observed that the sorptive saturation for all targets was attained within a 5 L sample volume loaded through the sorbent bed. Hence, the concentrations of each target species were first expressed as the ratio between those exiting and entering the sorbent bed such as [C_O_]/[C_I_]. These values were then plotted against loaded volume of HS sample (Figure 1S).

**Figure 2:**
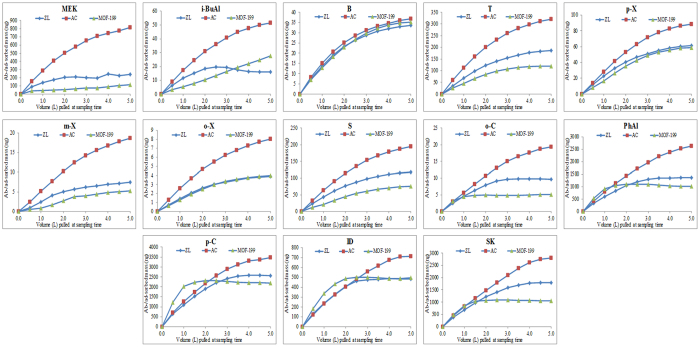
The relationship between the mass of analyte sorbed on surface (ng) vs. volume (L) pulled through sorbent materials.

To evaluate the sorptive behavior of the target compounds, the breakthrough volume (BTV) was computed for each odorant^35^. The 5, 10, and 50% BTV ([C_O_]/[C_I_]) values for the three sorbents were calculated and are shown in Table 2SA. Most of the target compounds attained 5 and 10% breakthrough within the first 0.5 L sample loading volume for all adsorbents (except PhAl, p-C, ID, and SK at 0.6, 0.6, 0.8, and 0.7 L, respectively for MOF-199). The 50% breakthrough occurred quite early for zeolite and MOF-199 sorbents. In contrast, activated carbon attained much larger values for the 50% BTV (e.g., o-X at 5 L). To determine the actual onset of the sorbent saturation with respect to loaded mass of odorants, total adsorbed mass (ng) was plotted against the amount of sample (volume (L)) pulled through the sorbent beds (Fig. 2).

### Adsorption isotherms of the odorants

An adsorption isotherm is a quantitative relationship describing the equilibrium between the concentration of adsorbate and its adsorbed concentration^36^. The adsorption isotherm at a specific temperature can be used to explain the relationship between the extent of adsorption and its parallel density. It is important to explain how adsorbates will interact with an adsorbent to critically optimize the use of adsorbent^37^.

The empirical Freundlich model is known to be a satisfactory approximation for sorption on a heterogeneous surface at low concentrations^38^. Hence, to interpret the adsorption equilibrium of the odorants using MOF-199 and the two reference adsorbents (ZL and AC), the equilibrium adsorption data obtained at 25 °C were analyzed by Freundlich model^39^:


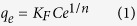


where, K_F_ and n are Freundlich constants related to the adsorption capacity and adsorption intensity, respectively. The Freundlich expression is an exponential equation. Therefore, it assumes that the concentration of adsorbate on the adsorbent surface increases with the increase of the adsorbate concentration. These parameters can be calculated from the intercept and the slope of the linear plot of log q_e_ versus log C_e_ using the following formula:


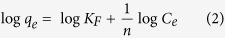


where the magnitude of the exponent (n) indicates the favorability of adsorption.

In our study, adsorption experiments were conducted at 298.15 K for VOCs adsorbed onto the adsorbents. Table 3 summarizes the parameters related to the Freundlich isotherms of the odorants for different sorbent materials. We observed high K_F_ and n values for phenolic and indolic compounds when compared to other VOCs. However, MOF-199 showed the highest adsorption performance for the phenolic and indolic compounds (R^2^ > 0.94), indicating a positive correlation between the odorant and the adsorbate concentrations. It can be anticipated that several factors govern the adsorption of phenolic and indolic compounds by MOF-199. Probably the most important factor is related to general, unspecific interactions, which come from the relationship between the pore and molecular sizes^40^. The interactions of the framework or the extra-framework species and hydrogen bonding between the hydrogen atoms of the adsorbate and the oxygen atoms of the framework might also be considered to be important^41^ and our data give some support to this theory.

### Adsorption capacity of MOF-199 for all target odorants

Partition coefficient (PC) is an important parameter to explain adsorption behavior between two interacting phases (viz., adsorbent and gas). As such, it can be used to characterize adsorbent heterogeneity and adsorption affinity of the odorants^42^,^43^. Therefore, as a means to assess the removal efficiency of the odorants released from slurry samples, we computed the PC (mmol kg^−1^ Pa^−1^) between each adsorbent and the odorants generated from slurry HS.

The PC values at 10% BTV for each odorant with respect to each adsorbent is summarized in Table 2S. MOF-199 showed highest PC values for o-C, PhAl, p-C, ID, and SK, whereas AC had the highest PC values for the other odorants (MEK, i-BuAl, B, T, p-X, m-X, and S). The enhanced PC values indicate higher adsorption due to enhanced affinity of the odorants to the adsorbent materials^44^. Table 4 shows a comparison of the odorant PC between this study and others. A noticeable difference in PC values was observed between our study and others^45^,^46^. However, the higher PC values in our study can be explained due to very low exit pressure^47^.

Table 4 also presents comparison of sorption capacities (μg g^−1^) between this study and those reported previously from other studies. To describe more detail about adsorption performance, we computed adsorption capacity (μg g^−1^) of each sorbent material at 5, 10, and 50% BTV.

The adsorption capacity of the zeolite, activated carbon, and MOF-199 are listed in Table 2S. Adsorption capacity, if expressed for each sorbent (zeolite, AC, and MOF-199) at 10% BTV for the sum of all 13 odorants was computed as 0.70  ±  1.08, 11.0  ±  18.3, and 22.6  ±  42.3 μg g^−1^, respectively. However, when the results for each odorant are compared, there were large differences in the maximum capacity measured for MOF-199 and AC. MOF-199 showed the largest adsorption performance for o-C (0.31  ±  0.04), PhAl (61.6  ±  4.98), p-C (140  ±  7.95), ID (27.8  ±  2.23), and SK (63.9  ±  1.55), while AC showed the maximum for MEK (5.02), i-BuAl (0.36), B (0.54), T (3.89), p-X (0.45), m-X (0.08), o-X (0.06), and S (1.57 μg g^−1^). The highest adsorption capacity for phenolic and indolic compounds observed for MOF-199 may be due to high surface area and presence of a large number of un-coordinated copper (II) sites, known to be as Lewis acids, demonstrating their efficacy as adsorption sites^23^. AC showed the best adsorption for the aromatic compounds compared to zeolite and MOF-199 because of the nonpolar nature of its surface^48^. However, AC showed the highest adsorption performance for almost all VOCs at 50% breakthrough.

The enhanced performance of AC and MOF-199 may be due to their very high micro-porosity and strong hydrogen-bonding between odorant molecules and the oxygen groups of the sorbent surface^20^,^49^,^50^. In contrast, the zeolite material showed the poorest performance for removing odors under our experimental conditions. The pore size and structural properties of zeolite are uniform and polar, while the aromatics are weak polar molecules. Hence, the adsorption of odorants by zeolite should not be affected significantly by molecular sieving effects as was seen for MOF^45^. Moreover, the lower adsorption observed for the zeolite is also assumed to be due to the effect of water vapor emitted from the impinger system as the zeolite can act as a water moderator. The large cavities and entry channels of zeolites can be filled with water molecules, forming hydration spheres around the exchangeable cations^51^. However, in our study the adsorption capacity of the phenolic and indolic compounds was noticeably higher for MOF-199 when compared to other VOCs. This may reflect the effect of strong hydrogen-bonding between the (−OH) and the (−NH) groups with the oxygen groups of the sorbent surface^52^,^53^,^54^.

The thermostability of the materials is determined by performing thermogravimetric analysis (TGA) of the fresh and used MOF samples. Figure 2S(A) indicates the TGA pattern of the fresh sample, while the results shown in Figure 2S(B–D) demonstrate the patterns obtained for used samples that were consecutively run for adsorption of slurry odorants. About 10% weight loss is seen below 100°C which could be attributed to the evaporation of solvent molecules adsorbed in the pores of MOF. After that point, a plateau was observed from 100 to 310 °C, indicating the maintenance of the structure within this temperature range. However, a further rise in the temperature resulted in a sharp weight loss demonstrating the collapse of the structure. The TGA pattern of the used samples presented in Figure 2S(B–D) tends to be fairly correlated with that of the fresh one. This indicates that the structure of the MOF is still intact even after the adsorption of odorants emitted from slurry. Based on this, we assume that the adsorption of odorants on MOF-199 is physical rather than chemisorption. Hence, the MOF-199 is sustainable and can be used efficiently for odorants/VOC removal from air.

## Conclusion

In this study, a simple dynamic impinger-based headspace sampling (HS) system was used to generate steady-state emissions of vapor-phase odorants and volatile organic compounds from a liquid slurry sample. The relative ordering of odorants emitted from the HS-sampling was: p-C (589 ± 4.23) > MEK (313 ± 15.0) > PhAl (272 ± 9.30) > SK (188 ± 0.00) > T (104 ± 12.1) > ID (67.7 ± 0.00) > S (53.4 ± 10.8) > B (32.1 ± 7.40) > p-X (29.7 ± 8.81) > i-BuAl (17.2 ± 0.00) > m-X (3.14 ± 0.21) > o-X (1.97 ± 0.52) > o-C (1.73 ± 0.00 ppb). The emission rates for most odorants decreased with increasing sample storage period, whereas relatively constant emission rates were observed for phenolic and indolic compounds due to their increased solubility.

In the second stage, the sorptive loss rate of different odorants was evaluated for MOF-199 and two reference sorbents (ZL and AC). The performance of the sorbent materials in removing the odorants was then compared in various respects. MOF-199 showed the best adsorption performance (at 10% BTV) for VOCs with high molecular weights (o-C (0.31 ± 0.04), PhAl (61.6 ± 4.98), p-C (140 ± 7.95), ID (27.8 ± 2.23), and SK (63.9 ± 1.55)), while AC showed the best for lighter compounds like MEK (5.02), i-BuAl (0.36), B (0.54), T (3.89), p-X (0.45), m-X (0.08), o-X (0.06), and S (1.57 μg g^−1^). These findings suggest the possible important role of MOF-199 as sorptive media for phenolic and indolic compounds. However, there remains significant scope for additional study to elucidate its interaction with various odorants under different environmental conditions (e.g., varying humidities). The effect of impurities on both the structure of adsorbent and on the binding affinity of the target adsorbate should also be investigated. In summary, our results open up an inquiry indicating new area of research for MOF-199 as a dynamic adsorption media for treating nuisance VOCs.

## Materials and Methods

### Instrumental setup and preparation of standards and slurry samples

In this study, a three-bed sorbent tube (ST) was employed for collecting the samples obtained from impinger-based dynamic headspace. To prepare the STs, quartz tubes were packed with three types of sorbent (50 mg of each) in the following order (from weakest to strongest in direction of sample flow): Carbopack C (60/80 mesh), Carbopack B (60/80 mesh), and Carbopack X (40/60 mesh); these three sorbents were separated and held in place with quartz wool. The sorbents were purchased from Supelco, USA. Before use, the STs were conditioned for 6 h at 320 °C by purging 99.999% N_2_ (flow rate = 100 mL min^−1^) through the STs using a tube conditioner (ATC-1200, ACEN Co. Ltd., Korea). The detailed procedure for preparing these STs is presented elsewhere^55^. Note, that the reliability of multiple-bed STs for the collection of various volatile compounds has also been validated in some recent studies^56^,^57^,^58^.

For an ST-based calibration, liquid standards containing all 19 targets (butyraldehyde (BA), methyl ethyl ketone (MEK), isovaleraldehyde (IA), valeraldehyde (VA), methyl isobutyl ketone (MIBK), butyl acetate (BuAc), isobutyl alcohol (i-BuAl), benzene (B), toluene (T), p-xylene (p-X), m-xylene (m-X), o-xylene (o-X), styrene (S), phenol (PhAl), p-cresol (p-C), indole (ID), and skatole (SK)) were prepared at seven different concentration levels (C1–C7: approximately 10, 20, 50, 100, 200, 500, and 1000 ng μL^−1^ for each) by dilution using methanol. All reagents were purchased from Sigma–Aldrich, USA and were used without further purification. PhAl, p-C, ID, and SK are solid powders, while the rest are liquids under ambient conditions. The solid phase chemicals were first dissolved in methanol to make the total solution of 20 mL. The explanation of the chemical acronyms in our study and their basic characteristics is presented in Table 1S. Different aliquots of the reagents were then used to prepare liquid working standards for a 7 point calibration (Table 3S).

In order to simulate VOCs from pigpen slurry, we collected slurry samples from a hog farm facility (with a size of approximately 60,000 m^2^) located in the Chungnam province in South Korea. The hog farm consists of various buildings including a pigpen (windowless and open) and two types of treatment facilities (compost and liquid treatment). In brief, the slurry wastes from the pigpen are sent through a pipeline to a tank for internal treatment and then separated into solid and liquid phases via a solid-liquid separator. The treatment of liquid slurry waste involved both aerobic and anaerobic processes. In this study, the liquid slurry samples collected from slurry treatment facility (before aeration) were used for assessing removal of the target odorants released, using different adsorbent materials. In our recent study, we intended to measure a broad range of odorants (e.g., nitrogenous compounds, organic sulfur compounds, and volatile fatty acids) to expand a profile of odorant emissions from swine facility^59^. In the current research, we focused on a total of 19 different VOCs (presented in Table 1S) to learn more about odor removal process, as most of them have been identified to be produced most dominantly from common pig farm facilities^15^,^16^,^17^.

### Selection of adsorbent materials for the removal of odorants emitted from pigpen slurry

Metal Organic Frameworks (MOFs) have become a novel field of research, resulting in numerous publications in the recent years^60^,^61^.

Adsorption is an important surface phenomenon and at the same time a common mechanism for the removal of both organic and inorganic pollutants^62^,^63^. MOFs have become popular to treat odors due to their effectiveness in adsorbing gaseous odors, while other common sorbents (e.g., zeolite (ZL) and activated carbon (AC)) have long been used for such purposes^63^,^64^,^65^,^66^,^67^,^68^

In this research, we selected MOF-199 and two other, more traditional sorbent materials (ZL and AC) to test the removal of the volatile odorants emitted from slurry samples. Zeolites have drawn a great deal of attention as potential sorbents due to their uniform micro pores and large internal surface area throughout their alumino-silicate crystal structure. It consists of 3-dimensional molecular-sized pores and channels running perpendicular to each other in the x, y, and z planes, while being made of secondary building units with large cavities^69^. AC, being economically most favorable, is used widely as adsorbent material for the removal of pollutants due to its micro-porous structure and high surface area^20^.

**Figure 3:**
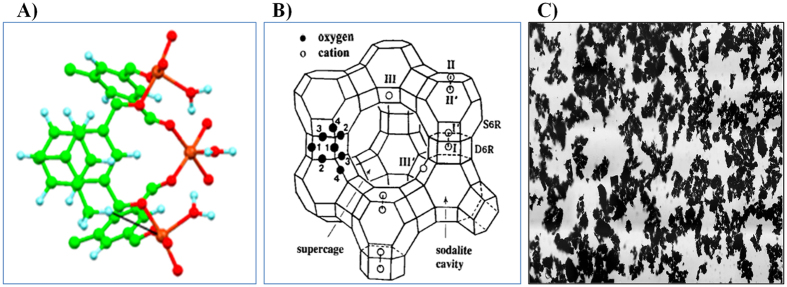
Structures of MOF-199, Zeolite (ZL), and Activated Carbon (AC): (**A**) Atomic structure of MOF-199; adapted with permission from^71^. Copyright (2016) Creative Commons Attribution 4.0 International; (**B**) Stylized drawing of the framework structure of ZL; adapted with permission from^73^. Copyright (2007) American Chemical Society; and (**C**) A bright field micrograph of AC; adapted with permission from^74^. Copyright (2010) Creative Commons Attribution-ShareAlike 3.0 Unported.

Synthetic Zeolite (A-4 bead) and granular AC were purchased from the manufacturers (Waco Pure Chemical Industries Ltd., (Japan) and Duskan Pure Chemical Co. Ltd., (South Korea), respectively). The MOF-199 sample was synthesized in the laboratory following the procedures reported elsewhere^70^. The synthesis procedures of MOF-199 can be described briefly as follows: 10 g of Cu(NO_3_)_2_·2.5H_2_O and 5 g of 1,3,5- benzenetricarboxylic acid (H_3_BTC) were stirred for 10 minutes in 250 mL of solvent consisting of N, N-dimethylformamide (DMF), ethanol, and water (1:1:1) in a 1 L jar, to form a slurry. The vessel was then heated at 85 °C for 20 hours. The blue crystals were washed with DMF and exchanged with dichloromethane (CH_2_Cl_2_) three times in three days. The dark blue crystals were then filtered and evacuated at 170 °C for 24 hours to give a final sample. The basic characteristics of the adsorbents used in our study are presented in Table 1. The basic structures of MOF-199, ZL, and AC are presented in Fig. 3. The XRD pattern of synthesized MOF-199 is presented in Figure 3S, whereas scanning electron microscopy (SEM) and Fourier transform infrared spectroscopy (FTIR) analysis results are discussed elsewhere^71^. The removal of odorants by the three sorbents tested in this work (MOF-199, zeolite, and activated carbon) should take place by a three stage adsorption mechanism as (i) diffusion to adsorbent surface, (ii) migration into pores of adsorbent, and (iii) monolayer buildup of adsorbate^72^.

### Experimental procedures for sorptive removal test

**Figure 4:**
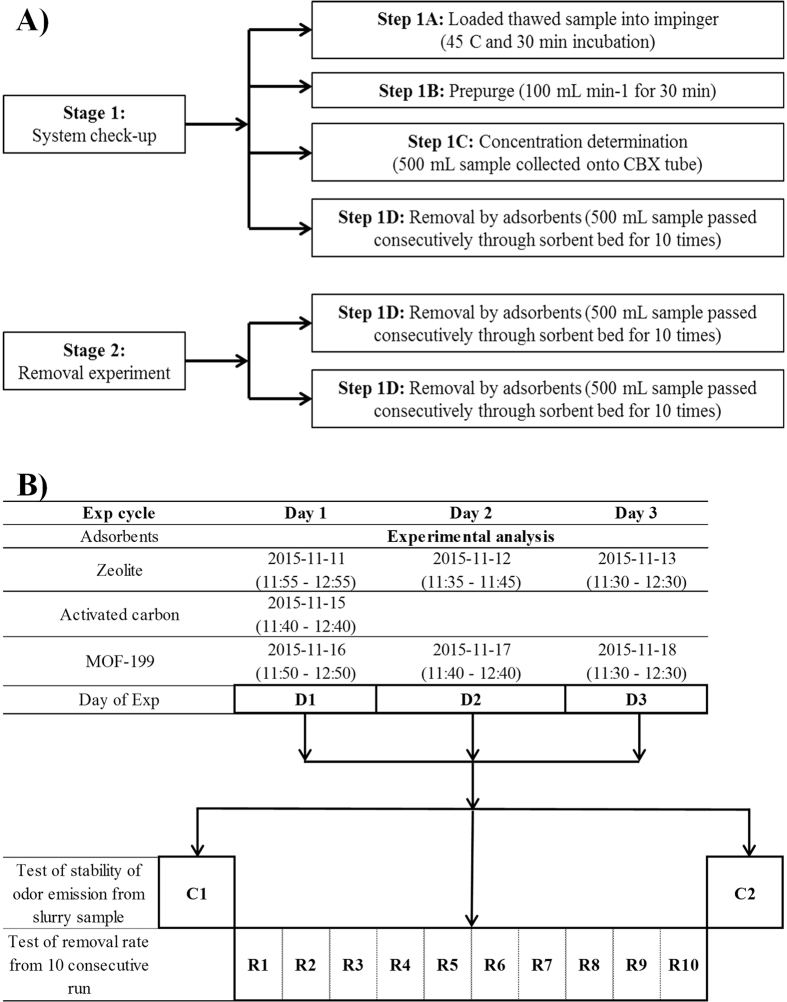
Schematic of the experimental design for the quantification of odorants emitted from pigpen slurry and their removal by three different sorbent materials (**A**) Basic flow chart for the quantification and removal of odorants emitted from slurry and (**B**) Schematic of the experimental analysis and data collection.

The odor removal experiments in this work were classified into two different stages. The first stage relates to the physical treatment employed for generating high and constant concentration odors from pigpen slurry samples and their quantification. The second stage is to measure their removal by different sorbent materials. The overall experimental scheme of this study is depicted in Fig. 4A. As the first stage of our experiment, a simple dynamic headspace (HS) sampling method made of impinger system with the capacity of 750 mL (Schott Duran, Germany) was employed to generate odorant emissions with constant and consistent concentrations from slurry samples. In brief, the gaseous sample was collected as follows: slurry samples previously stored in a refrigerator were thawed at room temperature for 14 h. Then, 25 g of slurry sample (P^H^ 7.8) was placed into a clean impinger with its temperature maintained at 45 °C. (Note that this temperature condition was chosen as it was suitable to cause emission of odorants from the sample at approximately ppb concentration levels). To collect the odorants, the following systems were connected in the order: a cylinder (containing 99.999% N_2_), an impinger, and finally a 3-bed sorbent tube (ST) (Figure 4SA). A pre-purge of sample in impinger was then made using a N_2_ flow (100 mL/min) for 20 min. Then 0.5 L of dynamic HS samples (flow rate of 100 mL/min for 5 min) was collected from the sample into a fresh ST to determine concentrations of the odorants initially emitted from slurry samples. The whole experiment was repeated on three consecutive days to assess the temporal stability of the odorant emissions with respect to storage time. A detailed schematic of the experimental analysis and data collection process is presented in Fig. 4B.

In the second experimental stage, MOF-199 (9.9 mg), zeolite (50 mg), and activated carbon (6.1 mg) were loaded into adsorption tubes to measure their removal efficiency by using 0.5 L dynamic headspace samples (5 min loading for each sorbent). Here, the same quartz tubes were used to prepare adsorption tubes packed with the three selected sorbents. This removal test was carried out ten times consecutively for a total loading volume of 5 L (total sampling time 50 min). Note that the masses of the adsorbents were chosen based on our preliminary experiments. The sorbent tubes filled with zeolite, activated carbon, and MOF-199 were connected to the outlet of the impinger and the other end was connected to a fresh 3-bed sampling ST (Carbopack CBX). The schematic of this set-up is presented in Figure 4SB.

## Additional Information

**How to cite this article**: Ahmed, E. *et al*. Performance comparison of MOF and other sorbent materials in removing key odorants emitted from pigpen slurry. *Sci. Rep.* **6**, 31283; doi: 10.1038/srep31283 (2016).

## Supplementary Material

Supplementary Information

## Figures and Tables

**Table 1:** Details of three types of adsorbents used for removal of the odorants emitted from slurry samples.

	Zeolite, Synthetic^a^	Activated Carbon^b^	MOF-199^C^
Adsorbent type	A-4 (bead)	Granular	Powder
Composition	1 Na_2_O: 1 Al_2_O3: 2.0 ± 0.1 SiO_2_ : x H_2_O	Charcoal activated	Cu_3_BTC_2_. xH_2_O
Diameter (mm)	1.40~2.36	—	—
Mesh size	8~12	4~14	—
Pore size (nm)	0.4	2	1.18
Pore volume (cm^3^ g^−1^)	4.1	0.48	0.693
Specific surface area (m^2^ g^−1^)	18.4	954	1781

^a^Manufactured by Waco Pure Chemical Industries Ltd.

^b^Manufactured by DUSKAN PURE CHEMICAL CO., LTD, and

^C^Synthesized in laboratory.

**Table 2:** Summary of the odorant concentrations and their emission rates (at a flow rate of 100 mL min^−1^) from pigpen slurry samples measured by an impinger-based headspace sampling.

Exp cycle^a^	Sample code^b^	MEK	i-BuAl	B	T	p-X	m-X	o-X	S	o-C	PhAl	p-C	ID	SK
Concentration (ppb)														
D1	C1	313 ± 15.0	17.2 ± 0.00	32.1 ± 7.40	104 ± 12.1	29.7 ± 8.81	3.14 ± 0.21	1.97 ± 0.52	53.4 ± 10.8	1.73 ± 0.00	272 ± 9.30	589 ± 4.23	67.7 ± 0.00	188 ± 0.00
	C2	165 ± 1.98	12.2 ± 0.59	7.37 ± 1.16	31.0 ± 1.91	12.7 ± 6.70	1.55 ± 0.31	0.68 ± 0.07	23.8 ± 9.35	1.73 ± 0.01	279 ± 2.06	588 ± 7.47	68.0 ± 0.46	191 ± 0.04
D2	C1	205 ± 3.24	9.92 ± 1.55	6.47 ± 2.27	42.0 ± 10.3	19.6 ± 7.87	2.55 ± 0.21	1.04 ± 0.01	31.4 ± 4.96	1.72 ± 0.12	293 ± 17.7	581 ± 5.17	74.4 ± 4.12	188 ± 8.96
	C2	105 ± 7.22	5.44 ± 0.27	0.78 ± 0.36	7.24 ± 3.05	4.54 ± 1.44	0.77 ± 0.10	0.27 ± 0.02	7.31 ± 0.84	1.75 ± 0.13	282 ± 6.98	583 ± 9.24	72.4 ± 4.20	184 ± 9.57
D3	C1	152 ± 12.7	6.18 ± 0.82	0.93 ± 0.63	13.3 ± 6.71	9.67 ± 3.60	1.56 ± 0.23	0.55 ± 0.00	14.0 ± 1.80	1.52 ± 0.15	282 ± 0.62	561 ± 10.9	84.8 ± 3.28	179 ± 6.46
	C2	79.4 ± 9.25	3.67 ± 0.66	0.27 ± 0.07	2.17 ± 1.45	2.25 ± 0.64	0.39 ± 0.01	0.14 ± 0.00	3.09 ± 0.03	1.53 ± 0.19	281 ± 1.39	581 ± 19.3	86.3 ± 6.98	179 ± 6.68
Emission rates (mg m^−2^ min^−1^)														
D1	C1	30.6 ± 1.46	1.72 ± 0.00	3.39 ± 0.78	13.0 ± 1.51	4.27 ± 1.27	0.45 ± 0.03	0.28 ± 0.07	7.52 ± 1.52	0.25 ± 0.00	34.7 ± 1.19	86.2 ± 0.62	10.7 ± 0.00	33.4 ± 0.00
	C2	16.1 ± 0.19	1.22 ± 0.06	0.78 ± 0.12	3.87 ± 0.24	1.83 ± 0.96	0.22 ± 0.05	0.10 ± 0.01	3.35 ± 1.32	0.25 ± 0.00	35.5 ± 0.26	86.1 ± 1.09	10.8 ± 0.07	33.9 ± 0.01
D2	C1	20.0 ± 0.32	1.00 ± 0.16	0.68 ± 0.24	5.24 ± 1.28	2.82 ± 1.13	0.37 ± 0.03	0.15 ± 0.00	4.42 ± 0.70	0.25 ± 0.02	37.4 ± 2.26	85.1 ± 0.76	11.8 ± 0.65	33.4 ± 1.59
	C2	10.2 ± 0.70	0.55 ± 0.03	0.08 ± 0.04	0.90 ± 0.38	0.65 ± 0.21	0.11 ± 0.01	0.04 ± 0.00	1.03 ± 0.12	0.26 ± 0.02	35.9 ± 0.89	85.4 ± 1.35	11.5 ± 0.67	32.7 ± 1.70
D3	C1	14.8 ± 1.24	0.62 ± 0.08	0.10 ± 0.07	1.66 ± 0.84	1.39 ± 0.52	0.22 ± 0.03	0.08 ± 0.00	1.97 ± 0.25	0.22 ± 0.02	35.9 ± 0.08	82.1 ± 1.60	13.5 ± 0.52	31.8 ± 1.15
	C2	7.75 ± 0.90	0.37 ± 0.07	0.03 ± 0.01	0.27 ± 0.18	0.32 ± 0.09	0.06 ± 0.00	0.02 ± 0.00	0.43 ± 0.00	0.22 ± 0.03	35.8 ± 0.18	85.0 ± 2.83	13.7 ± 1.11	31.8 ± 1.19

^a^D-1, 2, and 3 indicate 3 consecutive days (24 hour intervals).

^b^C2 was measured after 50 min of C1 (each concentration was measured by 500 mL of sample loading volume).

**Table 3:** Parameters of the Freundlich isotherms for the adsorption of odorants released from slurry samples by three different sorbent materials (MOF-199, activated carbon (AC), and zeolite(ZL)).

TargetCompounds	ZL			AC			MOF-199		
	n^a^	Kf^b^	R^2^^c^	n	Kf	R2	n	Kf	R2
MEK	−0.13 ± 0.16	31.8 ± 3.44	0.20 ± 0.07	0.42	0.00	0.08	−0.34 ± 0.05	965 ± 101	0.92 ± 0.02
i-BuAl	0.96 ± 0.31	0.03 ± 0.01	0.42 ± 0.09	0.16	0.00	0.42	−0.190.07	1442 ± 60.3	0.76 ± 0.08
B	−0.62 ± 0.09	14.9 ± 1.91	0.86 ± 0.07	−0.60	4.24	0.88	−0.58 ± 0.32	0.55 ± 0.39	0.93 ± 0.05
T	−0.52 ± 0.12	9.95 ± 1.01	0.88 ± 0.07	−0.42	2928	0.92	−0.71 ± 0.08	65.4 ± 6.84	0.91 ± 0.12
p-X	−0.44 ± 0.06	15.7 ± 2.63	0.83 ± 0.12	−0.44	137	0.92	−0.44 ± 0.15	38.7 ± 4.50	0.96 ± 0.05
m-X	−0.46 ± 0.13	0.12 ± 0.11	0.89 ± 0.01	−0.43	0.50	0.76	−0.63 ± 0.14	0.36 ± 0.27	0.85 ± 0.01
o-X	−0.44 ± 0.11	0.01 ± 0.02	0.79 ± 0.05	−0.45	0.02	0.94	−0.50 ± 0.07	0.22 ± 0.30	0.91 ± 0.08
S	−0.43 ± 0.09	14.2 ± 2.19	0.83 ± 0.11	−0.45	798	0.97	−0.54 ± 0.04	489.45 ± 18.6	0.96 ± 0.01
o-C	0.96 ± 0.05	0.12 ± 0.00	0.83 ± 0.09	0.71	2.23	0.81	3.44 ± 0.53	0.53 ± 0.02	0.91 ± 0.02
PhAl	0.76 ± 0.01	0.02 ± 0.00	0.85 ± 0.04	0.71	0.25	0.96	5.91 ± 1.36	39.7 ± 4.09	0.94 ± 0.13
p-C	0.54 ± 0.03	0.001 ± 0.00	0.86 ± 0.05	0.51	0.00	0.93	5.81 ± 1.44	73.3 ± 6.23	0.94 ± 0.02
ID	1.13 ± 0.12	0.23 ± 0.07	0.87 ± 0.03	0.84	1.17	0.91	5.55 ± 0.48	23.0 ± 2.66	0.98 ± 0.02
SK	0.95 ± 0.06	0.17 ± 0.05	0.92 ± 0.03	0.94	2.43	0.87	6.77 ± 0.98	49.6 ± 1.90	0.97 ± 0.01

^a^n is the Freundlich constant (adsorption power).

^b^Kf indicates the Freundlich constant (measure of the absorptivity of the adsorbent).

^c^R^2^ is the coefficient of the Freundlich equation.

**Table 4:** Comparison of partition coefficient (PC (mmol kg^−1^ Pa^−1^)), sorbent capacities (Cap (μg g^−1^)) and partial pressure (PP (Pa)) of VOCs for the stated adsorbents in different studies.

Parameters	Adsorbents	MEK	i-BuAl	B	T	p-X	m-X	o-X	S	o-C	PhAl	p-C	ID	SK	Reference
PC	AC	8.16	—a	—	20.4	—	—	—	—	—	—	—	—	—	46
Cap		200000	—	—	400000	—	—	—	—	—	—	—	—	—	
PP		341	—	—	213	—	—	—	—	—	—	—	—	—	
PC	AC	—	—	5.26	11.1	19.3	22.8	154	—	—	—	—	—	—	45
Cap		—	—	130000	87937	21618	22680	97000	—	—	—	—	—	—	
PP		—	—	508	171	130	133	131	—	—	—	—	—	—	
PC	ZL (13 X)	—	—	0.29	2.06	12.6	12.1	9.47	—	—	—	—	—	—	
Cap		—	—	5416	7920	14053	9555	5989	—	—	—	—	—	—	
PP		—	—	673	346	130	163	131	—	—	—	—	—	—	
PC	MOF—5	—	—	—	—	—	—	—	—	—	—	1100	1800	2900	71
Cap		—	—	—	—	—	—	—	—	—	—	1500	1400	2500	
PP		—	—	—	—	—	—	—	—	—	—	0.03	0.03	0.03	
PC	Eu-MOF			1200	1200	1100			1300		1300	2700	2700	5700	
Cap		—	—	1000	950	760	—	—	850	—	1500	3000	1500	3000	
PP		—	—	0.01	0.01	0.01	—	—	0.010	—	0.04	0.03	0.03	0.03	
PC	MOF-199			2100	5300	1100			1200		12000	17000	10000	20000	
Cap		—	—	>1100	>2600	>5200	—	—	>4900	—	13000	15000	4500	7700	
PP		—	—	0.01	0.01	0.01	—	—	0.01	—	0.04	0.03	0.03	0.03	
PC	ZL (A—4)	1.57	2.58	3.65	3.06	3.13	1.12	2.16	3.21	7.96	6.30	4.11	10.8	13.5	This study^c^
Cap		4.77	0.32	0.67	3.72	1.23	0.15	0.08	2.36	0.19	27.3	51.1	9.64	35.8	
PP		0−0.015	0−0.0007	0−0.0006	0−0.003	0−0.0008	0−0.0002	0−0.00006	0−0.002	0−0.00006	0−0.01	0−0.03	0−0.002	0−0.004	
PC	AC	40.8	44.9	98.4	93.4	35.3	34.9	60.7	52.5	93.7	118	46.0	126	218	
Cap		134	8.44	6.03	52.6	14.5	3.07	1.32	31.9	3.17	432	568	116	459	
PP		0−0.009	0−0.0004	0−0.0002	0−0.001	0−0.0006	0−0.0001	0−0.00003	0−0.001	0−0.00005	0−0.006	0−0.02	0−0.001	0−0.003	
PC	MOF-199	3.51	4.03	9.54	9.95	6.61	7.46	8.29	7.22	153	917	542	5865	4190	
Cap		11.8	2.78	3.56	12.1	5.93	0.53	0.40	7.59	0.51	103	221	50.1	106	
PP		0−0.02	0−0.001	0−0.0008	0−0.003	0−0.002	0−0.0002	0−0.00009	0−0.003	0−0.00002	0−0.0007	0−0.002	0−0.00003	0−0.0001	

^a^Not measured.

^b^Adsorption capacities after 15 L loading of gaseous standard.

^c^Adsorption capacities after 5 L loading of slurry odorants.
