# Supplementary material for: Performance comparison of MOF and other sorbent materials in removing key odorants emitted from pigpen slurry
Source: Sci Rep. 2016 Aug 11;6:31283. doi: 10.1038/srep31283 (PMC4980631; doi:10.1038/srep31283)
Supplement: Supplementary Information [file srep31283-s1.doc]

**Supporting Information**

**Performance comparison of MOF and other sorbent materials in removing key odorants emitted from pigpen slurry**

**Ezaz Ahmed^1^, Akash Deep^1,2*^, Eilhann E. Kwon^3^, Richard J.C. Brown^4^, Ki-Hyun Kim^1^***

^1^Department of Civil and Environmental Engineering, Hanyang University, 222 Wangsimni-Ro, Seoul 04763, Korea; ^2^Central Scientific Instruments Organisation (CSIR-CSIO), Sector 30 C, Chandigarh, 160030, India; ^3^Department of Environment and Energy, Sejong University, Seoul 05006, Republic of Korea; ^4^Environment Division, National Physical Laboratory, Teddington, TW11 0LW, UK

**Contents**

**Table 1S.** List of the 19 volatile organic compounds (VOCs) selected for sorptive removal for the three different sorbent materials (MOF-199, zeolite and AC)

**Table 2S.** Evaluation of breakthrough volume (BTV), adsorption capacity, and partition coefficient (PC) of the odorants at 5, 10, and 50% breakthrough points for different adsorbents

**Table 3S.** Preparation of liquid working standards (LWS) for the analysis by the TD/GC/MS system

**Table 4S.** Instrumental conditions for TD-GC-MS operation

**Table 5S.** Results of calibration analysis of the 19 selected compounds by the ST-TD-GC-MS method

**Figure 1S.** Normalized concentrations to assess adsorbent saturation (normalization was made with respect to [C_out_]/[C_in_])

**Figure 2S.** Thermogravimetric analysis of MOF-199 (A) fresh sample and (B, C, and D) the used samples.

**Figure 3S.** XRD patterns of synthesized MOF-199

**Figure 4S.** Overall scheme of the experimental setup used for sorptive removal test of volatile odorants

Correspondence: [dr.akashdeep@csio.res.in](mailto:dr.akashdeep@csio.res.in), Tel: +91-172-2657811-452, Fax: +91-172-2657287

[kkim61@hanyang.ac.kr](mailto:khkim@hanyang.ac.kr), Tel.: +1-82-2-2220-2325; Fax: +82-2-2220-1945

**Materials and methods**

**Quantitation of odorants and quality assurance (QA).** The analysis of all target compounds was made by using a thermal desorption (TD) unit (UNITY, Markes International Ltd., UK) interfaced to a GC (Shimadzu GC-2010, Japan) equipped with a mass spectrometric detector (Shimadzu GCMS-QP2010, Japan). The detailed operational parameters of the TD-GC-MS system is summarized in Table 4S. After being placed in the TD unit, the ST was heated to 300 °C and held for 5 min using UHP (99.999%) He gas. The targets were captured in a cryofocusing trap (Carbopack C and Carbopack B) at 5 °C, released by heating the trap to 300 °C for 5 min and separated on a polar column (CP-WAX; Varian, USA) under the conditions also given in Table 4S.

To perform calibration, 1 µL of each liquid working standard at seven different concentration levels (C1-C7) was injected onto the sorbent tubes (STs). To this end, the inlet of the ST was connected (via 2 cm long silicon tubing) to a 1 L polyester aluminum (PEA) bag filled with back-up UHP (99.999%) N_2_ gas; the outlet was connected to a mini vacuum pump (Sibata ΣMP-30, Japan). One microliter of the LWS was directly injected (using a 10 μL syringe, SGE Analytical Science, Australia) onto the internal wall of the ST by puncturing the silicon tubing connection (used as temporary injection port). At the same time, the back-up gas was introduced into the ST at a constant flow rate of 100 mL min^−1^ for 3 min to facilitate adsorption of analytes onto the sorbent materials. The results obtained by this calibration (Table 5S) were used to assess the concentration of the odorants emitted from slurry sample and to evaluate the removal efficiency of the sorbent materials.

The response factor (RF) values for the 19 selected targets varied from 13122 (BA) to 46864 (SK), while the coefficient of determinations (R*^2^*) were all well above 0.98. The performance of the ST-TD-GC-MS system was also evaluated via basic quality assurance (QA) parameters (Table 5S). As part of this, the method detection limits (MDL) and reproducibility (relative standard error (RSE)) of the ST-TD-GC-MS system were evaluated using the LWS at the lowest calibration point of ~5 ng µL^-1^. The MDL values were calculated following the US-EPA guidelines, i.e., the product of standard deviation (SD: n = 7 replicates) and the student's t-value at the 99% confidence level (t = 3.14 for 6 degrees of freedom). The MDL derived in terms of absolute mass ranged from 0.45 (o-X) to 2.28 ng (m-C). The RSE values (%) were found to vary in a range of 0.98 (m-X) to 4.64 (ID).

**Table 1S.** List of the 19 volatile organic compounds (VOCs) selected for sorptive removal for the three different sorbent materials (MOF-199, zeolite and AC)

| Full name | Short name | Formula | CAS number | MW (g mol^-1^) | Density (g mL^-1^) | Quantification ion | Carbon number |
| --- | --- | --- | --- | --- | --- | --- | --- |
| Butyraldehyde | BA | C_4_H_8_O | 123-72-8 | 72.1 | 0.81 | 41, 42, 43, 72 | 4 |
| Methyl ethyl ketone | MEK | C_4_H_8_O | 78-93-3 | 72.1 | 0.81 | 43, 72 | 4 |
| Isovaleraldehyde | IA | C_5_H_10_O | 590-86-3 | 86.1 | 0.80 | 41, 43, 44 | 5 |
| Valeraldehyde | VA | C_5_H_10_O | 110-62-3 | 86.1 | 0.81 | 41, 44 | 5 |
| Methyl isobutyl Ketone | MIBK | C_6_H_12_O | 108-10-1 | 100 | 0.80 | 41, 43 | 6 |
| Butyl acetate | BuAc | C_6_H_12_O_2_ | 123-86-4 | 116 | 0.88 | 41, 43 | 6 |
| Isobutyl alcohol | i-BuAl | C_4_H_10_O | 78-83-1 | 74.1 | 0.80 | 41, 42, 43 | 4 |
| Benzene | B | C_6_H_6_ | 71-43-2 | 78.1 | 0.88 | 78 | 6 |
| Toluene | T | C_7_H_8_ | 108-88-3 | 92.1 | 0.87 | 91, 92 | 7 |
| p-Xylene | p-X | C_8_H_10_ | 106-42-3 | 106 | 0.87 | 91, 105, 106 | 8 |
| m-Xylene | m-X | C_8_H_10_ | 108-38-3 | 106 | 0.87 | 91, 105, 106 | 8 |
| o-Xylene | o-X | C_8_H_10_ | 95-47-6 | 106 | 0.87 | 91, 105, 106 | 8 |
| Styrene | S | C_8_H_8_ | 100-42-5 | 104 | 0.91 | 78, 103, 104 | 8 |
| o-Cresol | o-C | C_7_H_8_O | 95-48-7 | 108 | 1.05 | 107, 108 | 7 |
| m-Cresol | m-C | C_7_H_8_O | 108-39-4 | 108 | 1.03 | 107, 108 | 7 |
| Phenol | PhAl | C_6_H_6_O | 108-95-2 | 94.1 | 1.07 | 94 | 6 |
| p-Cresol | p-C | C_7_H_8_O | 106-44-5 | 108 | 1.03 | 107108 | 7 |
| Indole | ID | C_8_H_7_N | 120-72-9 | 117 | 1.17 | 117 | 8 |
| Skatole | SK | C_9_H_9_N | 83-34-1 | 131 | 1.10 | 130, 131 | 9 |

**Table 2S.** Evaluation of breakthrough volume (BTV), adsorption capacity, and partition coefficient (PC) of the odorants at 5, 10, and 50% breakthrough points for different adsorbents

| **BT (%)** | **MEK** | **i-BuAl** | **B** | **T** | **p-X** | **m-X** | **o-X** | **S** | **o-C** | **PhAl** | **p-C** | **ID** | **SK** |
| --- | --- | --- | --- | --- | --- | --- | --- | --- | --- | --- | --- | --- | --- |
|  | **(A) BTV (L)** | | | | | | | | | | | | |
| **(I) Zeolite beads** | | | | | | | | | | | | | |
| 5 | 0.1 | 0.1 | 0.1 | 0.1 | 0.1 | 0.1 | 0.1 | 0.1 | 0.1 | 0.1 | 0.1 | 0.1 | 0.1 |
| 10 | 0.1 | 0.1 | 0.1 | 0.1 | 0.1 | 0.1 | 0.1 | 0.1 | 0.2 | 0.1 | 0.1 | 0.2 | 0.2 |
| 50 | 0.4 | 0.5 | 0.4 | 0.4 | 0.4 | 0.4 | 0.4 | 0.4 | 1.4 | 1.2 | 0.6 | 1.6 | 1.7 |
| **(II) Activated carbon** | | | | | | | | | | | | | |
| 5 | 0.1 | 0.1 | 0.1 | 0.1 | 0.1 | 0.1 | 0.1 | 0.1 | 0.1 | 0.1 | 0.1 | 0.1 | 0.2 |
| 10 | 0.1 | 0.1 | 0.2 | 0.2 | 0.1 | 0.1 | 0.2 | 0.2 | 0.2 | 0.3 | 0.1 | 0.3 | 0.4 |
| 50 | 1.3 | 2.0 | 4.5 | 4.8 | 3.6 | 4.1 | 5.0 | 4.5 | 3.0 | 2.8 | 0.9 | 2.9 | 3.8 |
| **(III) MOF-199** | | | | | | | | | | | | | |
| 5 | 0.03 | 0.02 | 0.04 | 0.04 | 0.03 | 0.02 | 0.04 | 0.03 | 0.3 | 0.5 | 0.5 | 0.6 | 0.6 |
| 10 | 0.1 | 0.1 | 0.1 | 0.1 | 0.1 | 0.1 | 0.1 | 0.1 | 0.5 | 0.6 | 0.6 | 0.8 | 0.7 |
| 50 | 0.3 | 0.3 | 0.4 | 0.4 | 0.3 | 0.2 | 0.3 | 0.3 | 1.0 | 1.2 | 1.2 | 1.5 | 1.3 |
|  | **(B) Adsorption capacity (ug g^-1^)** | | | | | | | | | | | | |
| **(I) Zeolite beads** | | | | | | | | | | | | | |
| 5 | 0.16±0.05 | 0.01±0.004 | 0.01±0.01 | 0.07±0.04 | 0.02±0.01 | 0.002±0.002 | 0.001±0.001 | 0.05±0.02 | 0.01±0.001 | 0.92±0.01 | 1.29±0.06 | 0.53±0.07 | 1.55±0.02 |
| 10 | 0.32±0.09 | 0.02±0.008 | 0.03±0.03 | 0.11±0.07 | 0.04±0.02 | 0.003±0.002 | 0.002±0.001 | 0.07±0.04 | 0.02±0.001 | 1.71±0.02 | 2.58±0.12 | 1.05±0.15 | 3.11±0.05 |
| 50 | 1.29±0.38 | 0.11±0.04 | 0.12±0.12 | 0.53±0.33 | 0.19±0.10 | 0.02±0.01 | 0.011±0.006 | 0.37±0.20 | 0.12±0.01 | 13.8±0.15 | 14.7±0.71 | 6.89±1.00 | 21.4±1.28 |
| **(II) Activated carbon** | | | | | | | | | | | | | |
| 5 | 2.51 | 0.18 | 0.27 | 1.94 | 0.23 | 0.04 | 0.03 | 0.63 | 0.10 | 13.8 | 13.8 | 4.36 | 29.6 |
| 10 | 5.02 | 0.36 | 0.54 | 3.89 | 0.45 | 0.08 | 0.06 | 1.57 | 0.19 | 34.6 | 27.7 | 9.90 | 59.1 |
| 50 | 58.8 | 5.08 | 5.89 | 51.9 | 13.0 | 2.79 | 1.32 | 30.7 | 2.46 | 306 | 187 | 89.0 | 412 |
| **(III) MOF-199** | | | | | | | | | | | | | |
| 5 | 0.25±0.08 | 0.01±0.02 | 0.08±0.06 | 0.22±0.21 | 0.05±0.03 | 0.01±0.002 | 0.01±0.003 | 0.08±0.03 | 0.17±0.02 | 56.2±4.36 | 123±4.11 | 21.4±2.29 | 56.4±1.68 |
| 10 | 0.42±0.13 | 0.04±0.04 | 0.16±0.11 | 0.49±0.47 | 0.10±0.06 | 0.02±0.01 | 0.01±0.005 | 0.16±0.06 | 0.31±0.04 | 61.6±4.98 | 140±7.95 | 27.8±3.23 | 63.9±1.55 |
| 50 | 2.52±0.78 | 0.22±0.18 | 0.81±0.56 | 2.07±0.97 | 0.47±0.27 | 0.07±0.02 | 0.04±0.02 | 0.78±0.31 | 0.43±0.08 | 97.9±8.99 | 214±25.9 | 43.9±5.43 | 97.1±1.56 |
|  | **(C) PC (mmol kg^-1^ Pa^-1^)** | | | | | | | | | | | | |
| **(I) Zeolite beads** | | | | | | | | | | | | | |
| 5 | 0.16 | 0.26 | 0.37 | 0.31 | 0.31 | 0.11 | 0.22 | 0.32 | 1.59 | 0.88 | 0.41 | 2.15 | 2.69 |
| 10 | 0.31 | 0.52 | 0.66 | 0.49 | 0.50 | 0.18 | 0.35 | 0.51 | 2.55 | 1.64 | 0.82 | 4.31 | 5.39 |
| 50 | 1.25 | 2.32 | 3.07 | 2.33 | 2.51 | 0.90 | 1.64 | 2.57 | 12.4 | 9.7 | 4.37 | 15.2 | 18.0 |
| **(II) Activated carbon** | | | | | | | | | | | | | |
| 5 | 4.08 | 5.39 | 19.7 | 18.7 | 3.53 | 3.49 | 9.71 | 6.30 | 18.7 | 23.6 | 5.51 | 27.7 | 87.3 |
| 10 | 8.16 | 10.8 | 39.4 | 37.4 | 7.05 | 6.98 | 18.2 | 15.8 | 37.5 | 59.1 | 11.0 | 63.0 | 175 |
| 50 | 93.8 | 156 | 1024 | 971 | 428 | 473 | 923 | 642 | 250 | 223 | 60 | 232 | 316 |
| **(III) MOF-199** | | | | | | | | | | | | | |
| 5 | 0.21 | 0.16 | 0.76 | 0.80 | 0.40 | 0.30 | 0.66 | 0.43 | 85.9 | 869 | 542.4 | 4755 | 3386 |
| 10 | 0.35 | 0.48 | 1.53 | 1.79 | 0.79 | 0.75 | 1.16 | 0.87 | 153.4 | 758 | 456 | 2535 | 2583 |
| 50 | 2.10 | 2.26 | 7.63 | 7.56 | 3.70 | 3.14 | 5.64 | 4.33 | 56.5 | 97 | 84 | 117 | 113 |

^a^Adsorption capacity was calculated as adsorbed mass / mass of adsorbent

^b^PC was calculated as adsorption capacity/P_out_

**Table 3S.** Preparation of liquid working standards (LWS) for the analysis by the TD/GC/MS system

**Table 4S.** Instrumental conditions for TD-GC-MS operation

| **(A). GC (SHIMADZU GC-2010, JAPAN), MS (SHIMADZU GCMS-QP2010, JAPAN)** | | | | |
| --- | --- | --- | --- | --- |
| Column: CP Wax (diameter: 0.25 mm, length: 30 m, and film thickness: 0.25 µm) | | | | |
| ***(1) Oven setting*** | |  | ***(2) Detector setting*** | |
| Oven temp | 40 °C (5 min) |  | Ionization mode | EI (70eV) |
| Oven rate: | 20 °C min^-1^ |  | Ion source temp: | 230 °C |
| Max oven temp: | 220 °C (16 min) |  | Interface temp: | 230 °C |
| Total time: | 30 min |  | TIC scan range: | 35-600 m z^-1^ |
|  |  |  | Scan speed | 1250 |
| ***(3) Carrier gas setting*** | |  |  |  |
| Gas type: | He (>99.999%) |  | Initial gas flow: | 1.03 mL min^-1^ |
| Constant gas pressure | 16.0 psi |  |  |  |
| **(B). Thermal desorber (Unity II, Markes, UK)** | | | |  |
| Cold trap sorbent: | Quartz wool + Carbopack C + Carbopack B + Quartz wool (Volume ratio = 1:1:1) | | | |
| Split ratio: | 0.093 |  | Adsorption temp: | 5 °C |
| Split flow: | 10 mL |  | Desorption temp: | 320 °C |
| Trap hold time: | 5 min |  | Flow path temp: | 180 °C |
| **(C). Sorbent (Sampling) Tube** | | |  |  |
| Sorbent material: | Carbopack C, Carbopack B, and Carbopack X (50 mg each) | | | |
| Desorption flow: | 100 mL min^-1^ |  |  |  |
| Desorption time: | 5 min |  | Desorption temp: | 300 °C |
| ^a^TD (UNIT, Markes International, Ltd., UK) | | | |  |

**Table 5S.** Results of calibration analysis of the 19 selected compounds by the ST-TD-GC-MS method

| **Short name** | **BA** | **MEK** | **IA** | **VA** | **MIBK** | **BuAc** | **i-BuAl** | **B** | **T** | **p-X** | **m-X** | **o-X** | **S** | **o-C** | **m-C** | **PhAl** | **p-C** | **ID** | **SK** |
| --- | --- | --- | --- | --- | --- | --- | --- | --- | --- | --- | --- | --- | --- | --- | --- | --- | --- | --- | --- |
| RF value | 13122 | 22067 | 19727 | 14454 | 22357 | 22970 | 24879 | 22669 | 38634 | 36815 | 43665 | 42194 | 40010 | 32244 | 38111 | 19422 | 30270 | 26913 | 46864 |
| R^2^ | 0.9967 | 0.9953 | 0.9932 | 0.9928 | 0.9969 | 0.991 | 0.9954 | 0.9918 | 0.9856 | 0.989 | 0.9917 | 0.9851 | 0.9876 | 0.9953 | 0.9806 | 0.9976 | 0.9929 | 0.9962 | 0.9969 |
| MDL (ng) | 0.43 | 0.74 | 0.72 | 1.26 | 1.41 | 1.12 | 1.72 | 1.06 | 1.30 | 1.35 | 1.36 | 0.45 | 1.38 | 1.58 | 2.28 | 1.37 | 1.28 | 0.78 | 0.86 |
| MDL (ppb) | 0.15 | 0.25 | 0.20 | 0.36 | 0.34 | 0.24 | 0.57 | 0.33 | 0.34 | 0.31 | 0.31 | 0.10 | 0.32 | 0.36 | 0.51 | 0.36 | 0.29 | 0.16 | 0.16 |
| RSE (%) | 4.20 | 2.10 | 4.28 | 1.95 | 1.21 | 3.73 | 1.89 | 1.81 | 1.89 | 2.23 | 0.98 | 1.59 | 3.79 | 4.48 | 3.02 | 4.25 | 3.27 | 4.64 | 2.81 |

**Figure 1S.** Normalized concentrations to assess adsorbent saturation (normalization was made with respect to [Cout]/[Cin])


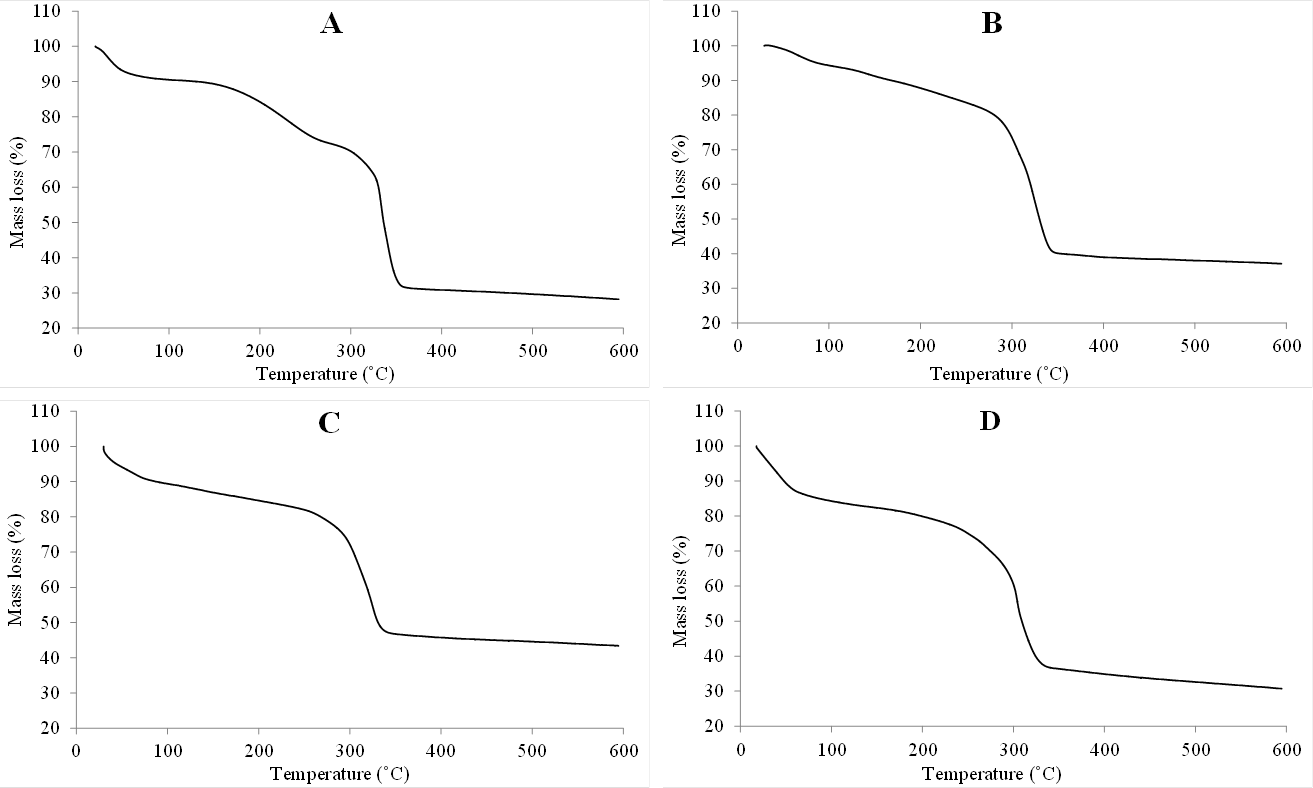


**Figure 2S.** Thermogravimetric analysis of MOF-199 (A) fresh sample and (B, C, and D) the used samples.

**Figure 3S.** XRD patterns of synthesized MOF-199

**Figure 4S.** Overall scheme of the experimental setup used for sorptive removal test of volatile odorants emitted from slurry
